# Early Pollen Sensitization in Children Is Dependent upon Regional Aeroallergen Exposure

**DOI:** 10.1155/2012/583765

**Published:** 2012-04-24

**Authors:** Vanessa Wong, Nevin W. Wilson, Kathy Peele, Mary Beth Hogan

**Affiliations:** Department of Pediatrics, University of Nevada School of Medicine, 343 Elm Street, Suite 206, Reno, NV 89503, USA

## Abstract

*Introduction*. Aeroallergen sensitization occurs at an earlier age than previously noted. The purpose of this paper was to identify which pollens cause early sensitization in young children presenting with rhinitis symptoms. *Methods*. This paper was a retrospective analysis of skin test results from 2- to 8-year-old patients presenting with a history consistent with allergic rhinitis. Patients were tested to aeroallergens common to the Great Basin along with a histamine and saline control. Pollen counts were obtained from a Reno, NV-certified counting station. *Results*. 123 children less than 8 years of age were identified. Over 50% of these children were sensitized to at least one aeroallergen. Chemopodaciae, timothy, alfalfa, black walnut, olive, mountain cedar and willow were predominating sensitizing aeroallergens of the Great Basin Region. Pollen counts were notable for a early spring peak for the tree season, grass season in May and weed season in August. Pollen levels continued to November at low levels. *Discussion*. Aeroallergens causing early sensitization differed from those which had predominately been reported in other regions of the United States. Pediatric allergists should consider performing a local review of sensitizing aeroallergens in their region to assist with identification and management of allergic rhinitis in their youngest patients. Please make style changes as appropriate.

## 1. Introduction

The prevalence of allergic diseases in childhood has increased considerably in developed countries over the last several decades [1]. Research has been published recently suggesting that children do in fact become sensitized to outdoor allergens at an early age and, thus, should be referred for skin testing earlier. One study found that outdoor allergens are as common sensitizing agents as indoor allergens by three years of age [2]. Another study found that the prevalence and incidence of seasonal allergic rhinitis increases after age two [3]. Furthermore, it has been found that sensitization to inhalant allergens occurring in the first few years of life is a major risk factor for developing persistent wheeze in childhood [4]. This study found that children with allergic rhinitis before the age of five were significantly more likely to have developed wheezing between age 5 and 13. They were nearly four times more likely to have childhood-onset wheezing. Children with early allergic rhinitis accounted for 41% of all cases of childhood wheezing.

The current research regarding outdoor allergen sensitization is limited to research conducted in eastern North America and other countries. There is little data concerning sensitization to allergens common to the western part of North America specifically the Great Basin of Nevada. This geographically unique region extends south along the entire Sierra Nevada Mountain Range from the Snake River Plain in southern Oregon to southern California and eastward to the Wasatch Mountain Range encompassing most of Nevada and a large portion of Utah with major cities of Las Vegas and Reno, Nevada, and Salt Lake City, Utah. Elevation ranges from near sea level to 13,000 feet. This is a closed drainage basin with rain and snow eventually leaving the basin by either evaporation, flowing into lakes (mostly saline), or sinking underground. This region is amongst the most arid regions in Continental North America.

The aim of this study was to identify in children the most frequent sensitizing pollen aeroallergens found in the Great Basin desert region. The identification of common sensitizers is relevant for both the identification and management of allergic disease, specifically allergic rhinitis and asthma in young children.

## 2. Methods

This study was a retrospective analysis of skin testing completed on 2- to 18-year-old patients who presented with a history consistent with allergic rhinitis conducted over 18 months from January 2009 ending June 2010. Patients were tested using commercially available extracts to common Great Basin outdoor allergens including weeds, grasses, and trees. The panel of common allergens was determined by consultation with the University of Nevada Agricultural Extension regarding frequency of allergenic plants native to the region and those upwind allergenic plants within 200 miles of the Great Basin to determine common pollens transferred to this region. In addition, local nurseries were called to determine which nonnative species were commonly sold.

Skin testing was performed using the prick-puncture method with a Greer DermaPik (Greer Laboratories, Lenoir, NC, USA) [5]. Saline and histamine controls were used in all patients. Test allergens producing a wheal of 3 mm or greater and at least half the size of the histamine wheal control were regarded as a positive reaction. False positive dermatographic saline controls were subtracted from all skin test results including the histamine before determining if the positive histamine size and positive skin test size were reached. The skin test results of 268 patients who met inclusion criteria were entered into an Excel spreadsheet for analysis. No unique identifiers were associated with the data collection, and the study met requirements for an IRB waiver.

Pollens relevant to the region included a possible panel of 10 weeds, 6 grasses, and 20 trees (Table 1). Pollen counts for the Great Basin Region correlating to the first continuous year of collected patient data were obtained from a Reno, NV-certified pollen counting station on the National Allergy Bureau (NAB) website. Pollen counts were recorded by the NAB as pollen grains/cumbic meter with trees at 1–14 low, 15–89 moderate, 90–1499 high and >1500 as very high. Grass pollen levels were recorded as 1–4 low, 5–19 moderate, 20–199 high. Weeds were noted to be 1–9 low, 10–49 moderate and 50–499 as high. The 2009 description of air-borne pollen levels in Reno, NV, USA are reported if changes in pollen counts are recorded at least two weeks in a row without change in level. Most common sensitizing plants were also noted if reported at least in two consecutive weeks. This is to avoid possible discrepancies induced by changing wind patterns.

## 3. Results

**Figure 1:**
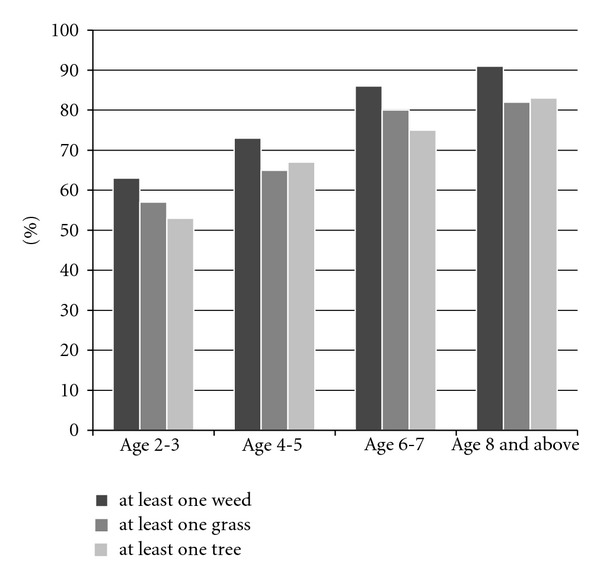
IgE reactivity to weed, grass, and trees of young children presenting with allergic rhinitis symptoms.

A total of 268 patients, ages 2–18 were included in this study. While all pediatric patients were initially included, it was noted that the sensitization rate for all trees, grasses, and weeds plateaued at age eight; 91% of children had a positive test to at least one weed, 82% were positive to at least one grass, and 83% were positive to at least one tree (Figure 1). In addition, the most common sensitizing tree, grasses, and weeds after age 7 were unchanged from the 6-7-year-old age group.

A total of 46% of the patients were under the age of eight years old, and this group was further defined due to observed differences in sensitization rates from children 8 years of age and older. Specifically, sixteen children in the age group 2-3 years, fifty-one children in the age group 4-5 years, and fifty-six children in the age group 6-7 years were evaluated.

In the age group 2-3 years, 63% of children tested positive to at least one weed, 57% of children tested positive to at least one grass, and 53% tested positive to at least one tree. As age increased, the percentage testing positive to at least one weed, one grass, or one tree also increased. The percentage of positive skin tests to outdoor allergens is seen in Figure 1 for each age group.

Pollen counting began in the Great Basin region at the start of the 2009 tree season in mid-January. Counts started at the low range mid-January and were observed in the high range by mid-February. Very high counts for trees became noted briefly in April and returning to the high range from May to end of June. Moderate levels of trees are noted in the Great Basin mid-July and fell to low counts noted mid-August. The tree season ended completely mid-November.

Grass season started with low counts in mid-April, moderate counts at the start of May and achieved briefly high counts at the end of May. Moderate counts in the 2009 season persisted to mid-July, when counts entered the low range consistently. Grass season ended at the end of September 2009.

Weed season started in the low range mid-May of 2009. Moderate counts were noted at the end of July. Weed pollen measurements dropped to the low range mid-Octorber and persisted in this range until the end of counting season in mid-November.

This 2009 season is notable in that all pollens were not generally noted to be in the very high range. However, the pollen season in the Great Basin is continuous from the beginning of consistent notation of low levels of tree pollen counted in January to the end of weed season. Tree season is extensive in length from mid-January to mid-November. Grass season is prolonged from mid-May to the middle of September. Weed season also is prolonged from mid-May to mid-November.

Not all pollens appear to sensitize young children. However, in the Great Basin Region, the Chenopodiaceae family, Timothy, and Alfalfa were frequently found. In addition, several trees such as walnut, olive, sweet gum, willow, and mulberry were frequently found. Thses sensitization patterns are outlined in Table 2 by age groups of 2-3 years, 4-5 years, and 6-7 years of age. The most common sensitizing allergenic plants were unchanged after 6 years of age. Sensitization to birch, cedar, mulberry, and maple was present in young children. Pine is not frequently thought to be a sensitizing tree but is found for prolonged lengths of time in the Great Basin region and is found to be early sensitizing tree pollen in our young children.

Sagebrush and pigweed were the most commonly noted weeds (Table 2). These weeds and their family members were reported consistently through the 2009 weed season. The ragweed family was not amongst the 3 most common weeds in this region even though this weed is the most commonly reported weed in the eastern half of the United States (east of the Rocky Mountain Range). Correlation of skin test sensitization frequency with the most prolific weeds in the Great Basin was seen in our young children and was notable for sagebrush and pigweed as the most frequently found sensitizing weeds. Skin test frequency for ragweed family was lower in these children than sagebrush and pigweed possibly a reflection of the lower ragweed pollen levels in the Great Basin.

## 4. Discussion

There is much debate as to whether children under the age of five are sensitized to outdoor allergens, and if so, which allergens are the culprits. In our study, we found that a large percentage of children were sensitized to at least one outdoor aeroallergen by the age of three. Reasons for this early sensitization in Westernized countries are unclear. Novel data in this study demonstrate that unlike other studies conducted in eastern North America, young children in the desert region of the Great Basin are sensitized to a different set of allergens. In fact, we found that young children living in the Reno area of the Great Basin are capable of being sensitized to allergens that were not previously appreciated to cause significant sensitization in this region at all.

In Washington DC, tree pollen accounted for 91.2% of the local total annual pollen production, while weeds and grasses accounted for only 3.8% and 3.2% of the pollen production respectively [6]. As such, in the eastern continental region of North America trees tend to be the common sensitizers of children with up to 56% of 10–12-year-old children becoming sensitized [7]. In a Boston area study, 17% of children fewer than 4 years of age were sensitized to trees. While lower than the sensitization rates achieved in the Great Basin, sensitization rates in Boston are still considerable. The Boston region likely has less aeroallergen sensitization than the Great Basin as it has the advantage of a much higer rate of rainfall. Available rain fall will likely shorten the allergy season by washing pollen away.

As our pollen data demonstrates, in the Great Basin Region of North America, unique geographic features are associated with prolonged detection of pollen in a more classic perennial fashion, rather than pollens solely acting as transient seasonal exposures. Features distinctive of the Great Basin include a lack of rainfall to remove pollen from the area and extremely windy conditions within a geographic bowl which cause pollens to become reairborne after settling on the desert floor. Due to these geographic factors, detection of some tree and grass pollen during the fall pollination season has been reported on a yearly basis albeit at low pollen levels. However, these low pollen levels were still potent causes of asthma exacerbations with increased asthma symptoms and rescue inhaler use [8]. Therefore, it is not surprising that in our area, trees and grasses were the pollens causing significant sensitization in very young children (53% and 57%, respectively). 

 Unlike the east coast in which ragweed tends to be the most common sensitizing weed, Russian thistle, pigweed, and sagebrush were the significant pollens causing early sensitization in the Great Basin. This is likely reflective of the fact that these weeds comprise a large portion of the plant population in our area. This is compared to ragweed being a more significant pollinating plant in the eastern and central plains of North America [9, 10].

While the weeds play a significant role in allergies in children living in the Great Basin, the grasses play almost an equal role. The prevalence of sensitization to at least one grass was lower than that of the weeds, but still was significantly high at 57% in children age 2-3 years old. The early grass sensitizers, Timothy and Alfalfa, are both grasses that are common to the Great Basin. Timothy grass is native to the region, and Alfalfa is planted for hay by cattle ranchers. Thus, it is not surprising that both were significant sensitizers in children. In our study, Brome was also a significant sensitizer which may reflect some cross-reactivity with Timothy. Similarly, the Great Basin native grass, Johnson grass, was a common sensitizer in the 2-3 years old. In our study, the Bermuda/Saltgrass family was a more common sensitizer in older children in the 4–7 age range.

Due to the inclusion of plants occupying western California which have the capability of spreading downwind, there were multiple nonnative plants included in our skin test panel. The best example of this is the pollen of the black walnut. Walnut was the most significant sensitizing tree allergen for children ages 2-3 years old which is surprising since walnut is not native to the Great Basin. Walnut is found in abundance in California, but this aeroallergen is capable of traveling great distances suggesting that it would be likely for California walnut pollen to be wind-borne to the Great Basin [11].

While walnut is a key sensitizer; olive also plays a large role in sensitization of children. Olive is a non-native plant included on our panel just as walnut is. However, members of this family, ash and privet are frequently transplanted and then irrigated by homeowners. In fact, privet has previously been implicated in olive-free regions [12]. It is not surprising that olive is a significant sensitizer in young children. In studies that have been done in the Mediterranean where olive is very common, they have found that olive is the most potent allergen eliciting a Type I reaction [13]. Olive was the second most common allergen causing a reaction in young children in this study. This further substantiates the thought that olive is a powerful sensitizer even in young children. 

Previous research has suggested that pines are rarely allergen sensitizers due to their pollen size [14]. Recent studies, however, found that pine pollen is a potential allergen, especially in areas where pine is found abundantly [15, 16]. Pine is commonly found in the Great Basin and in our study was found to cause sensitization in children 16–25% of the time. While sensitization to pine is not as high as some of the other tree allergens, pine should not be overlooked as a potential allergen in young children living in regions with high prevalence of this tree.

Finally, willow, sweet gum, and mulberry are not native species to the area, yet they still cause sensitization in young children. A reason for this sensitization to nonnative species may be due to the transplantation of these trees to this area. In addition, one study suggests that willows are not important aeroallergens since they are pollinated via insects as opposed to the wind [14]. However, willow is also pollinated by wind-borne means and frequently found as a transplanted tree in Reno area. Another study found strong cross-reactivity among members of the family, *Salicales* with which willow, poplar, and cottonwood belong to [11]. In our study willow was a significant early sensitizer with a large prevalence in children ages 2-3 years, and this occurred in the absence of poplar and cottonwood suggesting that cross-reactivity was not the reason for IgE responses to willow. This suggests that willow's large presence in the Great Basin may account for its ability to provoke IgE sensitization response.

Our data suggests that many children with allergic rhinitis living in the Great Basin were sensitized to an outdoor allergen by as early as 2 years of age. This further supports previous publications demonstrating that children less than 5 years of age develop allergic rhinitis and deserve evaluation. Specifically, there are several weeds, grasses, and trees that are native to the Great Basin region including native, wind-borne, and transplanted species that cause significant sensitization leading to allergic rhinitis in these young children. Allergic rhinitis first appears in the preschool years and was found to be a predictor of the onset of wheezing later in childhood [17]. For preschool children with allergic rhinitis, assessment of allergic sensitization might help in identifying a group at high risk of wheezing and therefore may be candidate for allergy immunotherapy as a disease modifier. For these reasons, we believe that even young children patients with allergic rhinitis who live in the Great Basin should have allergy testing to the most likely pollens to each of the weeds, grasses, and trees that cause sensitization at an early age. More importantly, we encourage other pediatric allergists to determine which aeroallergens are early sensitizers in their region to help specifically plan targeted aeroallergen skin testing in these small but symptomatic allergic rhinitis and asthmatic children.

## Figures and Tables

**Table 1:** Great Basin aeroallergens available in skin test panel.

Weeds	Grasses	Trees	
Dock	Timothy	Privet	Birch
Pigweed	Brome	Mountain Cedar	Olive
Russian thistle	Bermuda	Sycamore	Ash
Saltbush	Saltgrass	Pine	Aspen
Sagebrush	Johnson	Elm	Willow
Western ragweed	Alfalfa	Ailanthus	Cottonwood
Tall and short ragweed		Locust	Pecan
Rabbitbush		Maple	Walnut
Marsh elder		Sweet gum	Alder
Plantain		Mulberry	Oak

**Table 2:** Most common aeroallergens by age group.

2-3-year-old children	Pollen	Positive percent of 16 children
Most common weeds	Russian thistle	60%
	Pigweed	58%

Most common grasses	Timothy	45%
	Alfalfa	56%
	Brome	40%
	Johnson	40%

Most common trees	Walnut	75%
	Olive	55%
	Mountain cedar	50%
	Willow	50%
	Sweet gum	33%
	Mulberry	43%
	Pine	25%

4-5-year-old children	Pollen	Positive percent of 51 children

Most common weeds	Russian thistle	54%
	Pigweed	46%
	Sagebrush	43%

Most common grasses	Timothy	42%
	Johnson	44%
	Alfalfa	37%

Most common trees	Olive	42%
	Maple	39%
	Willow	31%
	Sweet gum	23%
	Mulberry	29%
	Pine	19%

6-7-year-old children	Pollen	Positive percent of 56 children

Most common weeds	Russian thistle	68%
	Pigweed	61%
	Sagebrush	49%

Most common grasses	Saltgrass	58%
	Timothy	48%
	Bermuda	45%
	Johnson	45%

Most common trees	Willow	39%
	Sweet gum	32%
	Mulberry	36%
